# Hemodynamic Effects on Particle Targeting in the Arterial Bifurcation for Different Magnet Positions

**DOI:** 10.3390/molecules24132509

**Published:** 2019-07-09

**Authors:** Sandor I. Bernad, Daniela Susan-Resiga, Elena S. Bernad

**Affiliations:** 1 Centre for Fundamental and Advanced Technical Research, Romanian Academy—Timisoara Branch, Mihai Viteazul Str. 24, RO-300223 Timisoara, Romania; 2 Faculty of Physics, West University of Timisoara, Vasile Parvan Str. 1, RO-300222 Timisoara, Romania; 3 University of Medicine and Pharmacy “Victor Babes” Timisoara, P-ta Eftimie Murgu 2, RO-300041 Timisoara, Romania

**Keywords:** magnetic particle targeting, arterial bifurcation, particle accumulation, hemodynamics

## Abstract

The present study investigated the possibilities and feasibility of drug targeting for an arterial bifurcation lesion to influence the host healing response. A micrometer sized iron particle was used only to model the magnetic carrier in the experimental investigation (not intended for clinical use), to demonstrate the feasibility of the particle targeting at the lesion site and facilitate the new experimental investigations using coated superparamagnetic iron oxide nanoparticles. Magnetic fields were generated by a single permanent external magnet (ferrite magnet). Artery bifurcation exerts severe impacts on drug distribution, both in the main vessel and the branches, practically inducing an uneven drug concentration distribution in the bifurcation lesion area. There are permanently positioned magnets in the vicinity of the bifurcation near the diseased area. The generated magnetic field induced deviation of the injected ferromagnetic particles and were captured onto the vessel wall of the test section. To increase the particle accumulation in the targeted region and consequently avoid the polypharmacology (interaction of the injected drug particles with multiple target sites), it is critical to understand flow hemodynamics and the correlation between flow structure, magnetic field gradient, and spatial position.

## 1. Introduction

Peripheral arterial disease (PAD) consists of an obstruction to the blood flow in the arteries, and this affects the aorta, the common, external and internal iliac artery, common femoral and profunda, superficial femoral, popliteal, infrapopliteal (anterior tibial, posterior tibial, peroneal), and intrinsic pedal arteries.

Survival in patients with lower extremity PAD is low as a result of atherosclerotic complications in the coronary and cerebrovascular beds. Management of patients with lower extremity PAD requires a solid knowledge of risk and the complications associated with all treatment options and also the ability to recognize and manage procedural complications of the arteries’ revascularization [1].

Which type of revascularization treatment is used, and the success of treatment, depends on several factors like the type of lesion (stenosis or total occlusion), the location and length of the blockage, the amount of calcification, the amount of damage to the artery, and the patient’s overall health [2,3,4]. All medical solutions have both advantages and disadvantages. As an example, (i) catheter-based interventions for angioplasty carry lower risks compared to surgery and reduce the duration of hospitalization; (ii) stenting is limited by the recurrent problems of late target lesion revascularization and stent thrombosis [5]; (iii) in-stent neo-atherosclerosis and stent fractures are other important contributing factors for late adverse events, including late stent stenosis and late target-lesion revascularization [6].

Magnetic drug delivery has been proposed as a potential solution for the problems mentioned above, to improve both drug targeting and the long-term survival of a patient with lower extremity PAD [7,8,9].

The literature shows a multitude of studies dedicated to the investigated artery bifurcation flow and also targeting possibilities, including experimental [10,11,12,13], theoretical [14,15], and numerical analysis [16,17,18,19] associated with particle deposition in the artery lesion site.

This paper focuses on investigating the feasibility and efficiency of drug targeting in the arterial bifurcation region from the perspective of the correlation between hemodynamic characteristics and particle deposition efficiency using a constant magnetic field over time, generated by a single permanent magnet.

For example, Kong [20] et al. present the advantage of using gold nanoparticles (Au NPs) for various biomedical applications, including molecular imaging, drug carriers, and biosensing. The advantages of the application of the magnetic drug targeting for cancer therapy are presented in [21], where the authors describe the effectivity of in-house fabricated MTO-loaded SPION (mitoxantrone loaded iron oxide nanoparticles) for magnetic drug targeting in multicellular tumor spheroids. The study in [22] presents a detailed review of the clinical relevance of nanomedicine, both in oncology and cardiology. It is essential to mention the side effects of intravenous therapies with nanoparticle-containing drugs and biological side effects called complement activation-related pseudoallergies (CARPAs), described in detail in [23].

## 2. Problem Description

### 2.1. Investigated Arterial Bifurcation

**Figure 1:**
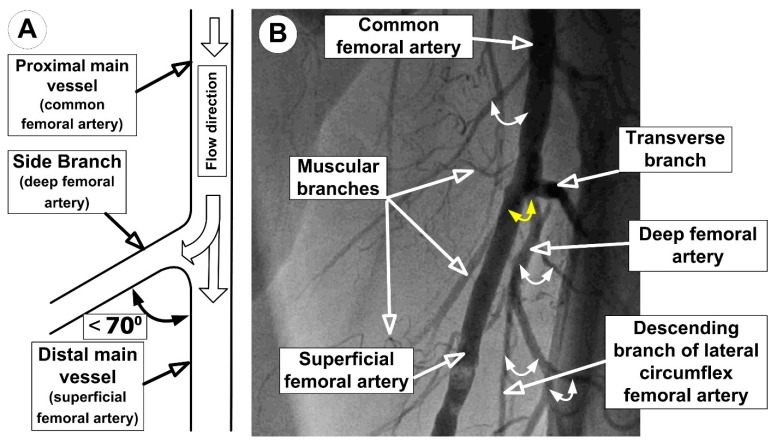
(**A**) Schematic representation of the arterial bifurcation. The yellow arrow indicates bifurcation between the superficial femoral artery and deep femoral artery. (**B**) An angiographic image of femoral artery bifurcations.

Bifurcation is a junction of the main vessel and a side branch. The bifurcation has three segments (Figure 1A): the proximal main vessel, the distal main vessel, and the side branch. The bifurcation angle is the angle between the axis of the most proximal part of the side branch and the axis of the distal main vessel. Based on this angulation, coronary artery bifurcations are classified into three groups [24]: 

1. Y-shaped angulation (acute angulation), in which the angle between the side branch and the distal main vessel is <70.

2. T-shaped angulation (right or near-right angulation), in which the angle between the side branch and the distal main vessel is 70–90.

3. Reverse-shaped angulation (obtuse angulation), in which the angle between the side branch and the distal main vessel is >90.

From a clinical point of view, Y-shaped angulation constitutes 76.1% of the arterial bifurcation lesions compared to 23.9% T-shaped angulation type lesions [24]. As shown in Figure 1B, branches formed by the femoral artery are of the Y-shape type. Anatomically, in the proximal part of the femoral triangle, the femoral artery gives off a branch, the deep femoral artery (Figure 1B). This arterial segment is called “the femoral bifurcation”. The superficial femoral artery represents the distal part of the femoral artery. The mean diameters for the common femoral artery are 10.6 ± 0.4 mm (range 8.2–12.7 mm), which is 58% larger than the superficial femoral artery, averaging 6.7 ± 0.3 mm (range 5.5–8.2 mm), and 76% larger than the deep femoral artery, averaging 6.0 ± 0.4 mm (range 4.5–8.0 mm) [25,26].

### 2.2. Arterial Bifurcation Lesion

**Figure 2:**
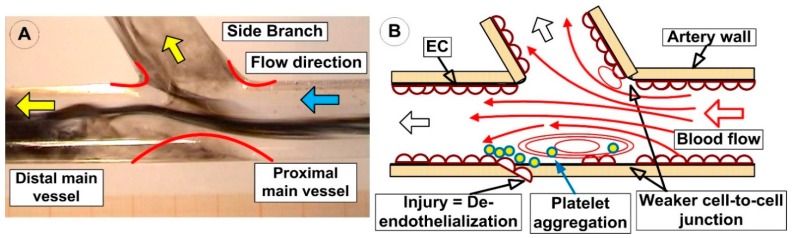
Flow hemodynamics of the artery bifurcation. (**A**) Development of the low wall shear stress (WSS) regions on the bottom wall of the main vessel in the opposite of the side branch, as well as along the lateral wall of the branch itself; (**B**) Flow bifurcation induces local vortices both in the distal main vessel and side branch and also creates a stagnation region with a long residence time for circulating particles. Flow deceleration and recirculation promote longer residence times, increased collision rates, and increased platelet aggregation. Low shear rates observed in these regions also favor the adhesion of platelets at the vascular wall. Platelets potentially activated by exposure to high shear and subsequently entrapped in vortices could potentially lead to the formation of free emboli.

A branching artery induces a significant effect on flow patterns in the main vessel in the vicinity of the bifurcation point [24]. In this region, secondary flow patterns can develop in the main vessel because part of the fluid is forced to change direction toward the side branch (Figure 2). 

As a result of these deviations away from the primary direction of the flow (from inside to the main vessel), regions of low wall shear stress (WSS) can develop on the bottom wall of the vessel in the opposite of the side branch, as well as along the lateral wall of the branch itself (Figure 2A).

Vessel bifurcations produce local vortices with increased mass transfer rates, and, as a result, this region has potential docking points for different circulating particles. Decreased blood flow in bifurcation section favors red blood cell (RBC) and platelet aggregation, especially in the locally generated vortices (characterized by enhanced mass transfer towards the vessel wall) [27,28].

Blood flow in the arteries induces the migration of various suspended particles (leukocytes, platelets, and rigid micro-particles) from the center of the vessel to the arterial wall [29,30,31]. This phenomenon represents the margination processes, which is a consequence of the competition between lift forces on RBCs and suspended particles and their interactions in the flow [32]. In general, in flowing suspensions, particles migrate inward from the vessel’s center to the vessel’s wall at a rate that increases with particle size [33].

### 2.3. Purpose of the Study

The present paper investigates the feasibility of using a single permanent magnet to trap circulating magnetic particles (Fe particles) within a bifurcating vascular system. This paper presents a single artery bifurcation targeting analysis to improve targeting efficiency. This methodology could be consecutively applied at several branches of the vascular network.

The present investigation used a macroscopic length scale for its useful experimental resolution. This scale relates directly to large and medium blood vessels (like the femoral artery) where blood hydrodynamics have a strong influence on particle depositions.

Magnetic particle targeting is done by analyzing the aggregate particles’ sizes, positions, and the influence of the magnetic field position on their deposition efficiency.

## 3. Materials and Method

### 3.1. Experimental Test Rig

**Figure 3:**
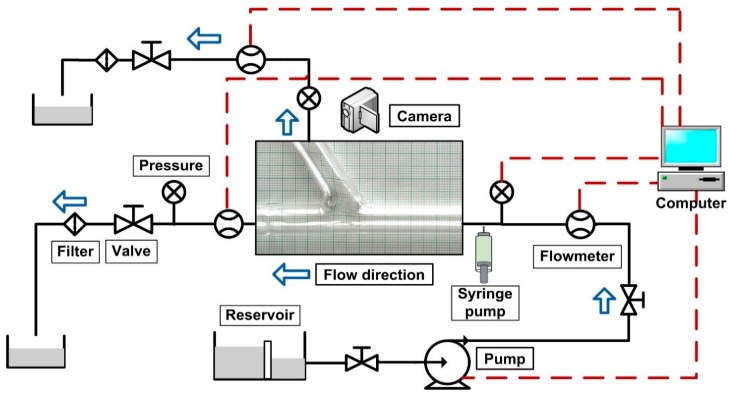
The experimental setup used for artery bifurcation investigations. The block diagram of the main recirculating flow loop contained a flowmeter, particle injection mechanism—syringe pump and test section—bifurcation model, valve, particle filter, reservoir, and centrifugal pump.

The experimental setup used for modeling the targeting of magnetic particles in the arterial bifurcation flow system is shown in Figure 3.

A computer-controlled pump (centrifugal pump GAMPT mbH, Germany) supplied a flow of carrier fluid (water-glycerin mixture) through a circuit of tubing to the artery bifurcation model (Figure 3). The magnetic particles (MPs) were released from a microliter syringe pump into a steady laminar main flow before the flow entered the bifurcation model’s inlet section. The injection volume was measured from the syringe pump’s readings. In the region of the artery bifurcation, a permanent ferrite magnet (26 mm × 26 mm × 5 mm) was placed under the tube in different vertical positions to establish the required magnetic field.

The measurement procedure used for the investigation was as follows. The main flow was turned on and adjusted up to 0.12 m/s, corresponding to a 362 mL/min flow rate. The position of the permanent magnet was adjusted to the desired distance from the bifurcation region wall using a sliding device to precisely position the permanent magnet. The syringe pump injected a steady stream of the carrier fluid containing MPs into the main flow at a bulk velocity of 0.12 m/s for a time of 30 s. The injected carrier fluid velocity was equal to the velocity of the main flow in the center of the graft tube.

Table 1 summarizes the parameters used in the experimental setup to visualize the particle trajectory. Particle distribution and flow field in the bifurcation region were investigated using the image analysis technique. To visualize flow dynamics and particle depositions, a Sony digital camera (XC-CCD series digital camera) was used to record the deposition processes [34].

Magnetic targeting methods used in this model rely on external magnets (permanent magnets), which are placed close to the artery bifurcation surface to provide the magnetic field strength and gradients necessary to concentrate magnetic entities in the specific region [35,36,37].

The parameters presented in Table 1 present the anatomical and biological conditions associated with the femoral artery bifurcation presented in Section 2.1 (the diameter of the femoral artery bifurcation [25,27] and the mean flow rate in the femoral artery [25,38]). Further, parameters indicated the suggested/used values in the literature for drug targeting studies (the size of the magnetic particles, the intensity of the magnetic field [36,37], and the viscosity of the analog blood fluid [39]).

### 3.2. Experimental Bifurcation Geometry

**Figure 4:**
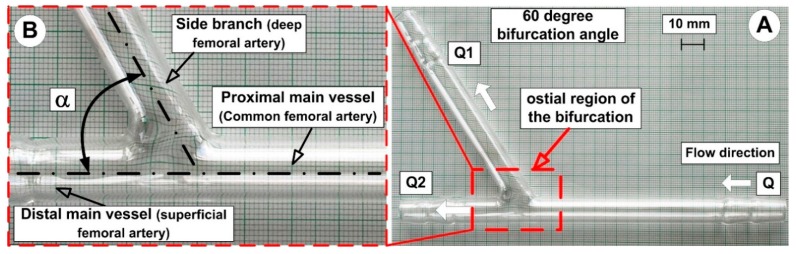
Experimental arterial bifurcation model. (**A**) General view of the model fashioned with a constant internal diameter of 8 mm. (**B**) Detail regarding model bifurcation angle. Notations used in this figure: PMV (proximal main vessel), DMV (distal main vessel), SB (side branch), α (bifurcation angle).

The test sections with bifurcation angles of 60° were designed to mimic the branching of the common femoral artery into the deep femoral artery and superficial femoral artery, fashioned into the right shape from glass tubing. The glass tube has a constant internal diameter of 8 mm both for the main vessel and the side branch (Figure 4). The flow rate ratios (Q1/Q2, Figure 4) were determined by two valves, one for the distal main vessel and one for the daughter branch. The diameters and flow ratios used were chosen to typically characterize the femoral artery flow [25,38].

All experiments used steady flow conditions and a realistic blood velocity measured in the human femoral artery bifurcation. Flow experiments were conducted under typical physiological conditions, where the mean flow velocity was Um = 0.12 m/s, corresponding to Reynolds Re = 283 [38].

It is important to keep in mind that this analysis is not meant to model the magnetic drug targeting accurately but is intended to find realistic ranges for a set of parameters that we will use in further simulations and experiments.

The glass used to mimic vascular vessels did not reproduce the mechanical and biological characteristics of real blood vessels, but using glass tubing provided several advantages. These advantages include high transparency of the vessel wall, which facilitates observation of the evolution of the hemodynamic characteristics, and the deposition process of the magnetic particle in the area of interest (measuring the deposition length and accumulation position).

### 3.3. Blood Analog Fluid Preparation

The working fluid (carrier fluid) used for this analysis was a blood analog fluid, which has a density (ρ) the same as the blood (1060 kg/m^3^). In this experiment, the flow was steady and contained only minimal regions with shear rates lower than 10 sec^−1^. Consequently, the blood viscosity model does not include viscoelasticity [39,40].

#### Rheological Properties of the Blood Analog Fluid

**Figure 5:**
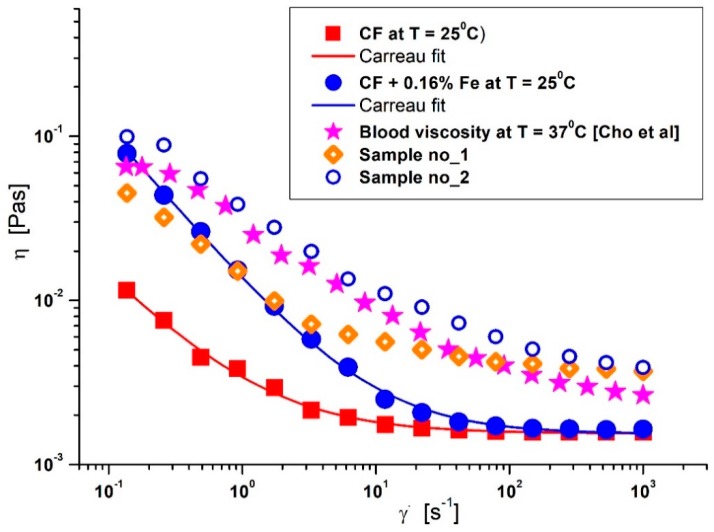
Comparison between the viscosity curves of the blood analog carrier fluid (glycerol–water fluid), the model suspension fluid (CF + 0.16% ferromagnetic particles), and blood. Blood viscosity curves were compared with values from the literature [41] and values measured from two healthy volunteers (Sample no_1 and Sample no_2).

For blood analog carrier fluid (CF), glycerol–water solutions were prepared by mixing calculated weights of distilled water and glycerol. The viscosity curves at T = 25 °C, of the carrier fluid presented in Figure 5 indicate a shear-thinning behavior (typical of pseudoplastic systems) at low shear-rates. This behaviour becomes Newtonian at shear rates >10 s^−1^.

In order to experimentally investigate the effect of blood flow on the different cardio-vascular pathologies, various blood-analog fluids are presented in the literature. The most common fluids used were glycerol–water solutions. The non-Newtonian characteristics of blood are due to the presence of hematocrit [41]. The experimental blood flow investigations aim to correctly reproduce the effect of non-Newtonian characteristics on the specific site of the coronary diseases, especially in the presence of the bifurcation region or the stenotic region where flow separation and a recirculation region appear. The used glycerol–water solution ensures the reproduction of the rheological behavior of the blood [41].

**Figure 6:**
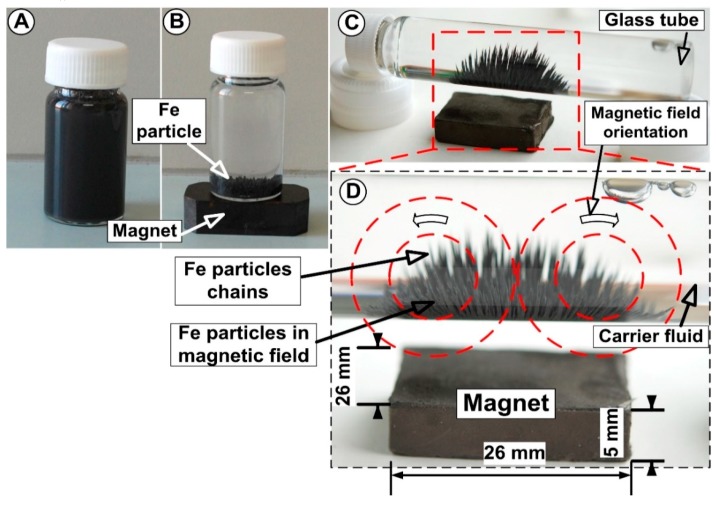
Ferromagnetic particle (FMP) separation in the presence of the magnetic field; (**A**) suspension in the absence of the magnetic field; (**B**) Fe particle separation in the presence of the magnetic field; (**C**) Fe particles contained in the model suspension forming chain structures aligning along the direction of the applied magnetic field. (**D**) Detail of the magnetic field orientation and and the ferromagnetic particle deposition along the magnetic lines.

In this paper, to evaluate targeting efficiency in the artery bifurcation region, ferromagnetic particles (FMP’s) were utilized to quantify the particle deposition on the vessel bifurcation. The model suspension of magnetic carriers used in the experiments was obtained by mixing blood analog carrier fluid (CF) (aqueous glycerol solutions) with iron (Fe) particle (4–6 μm in size, Carl Roth GmbH, Karlsruhe, Germany) with 0.16% mass concentration. The degree of change in the model suspension depends on the magnitude of the applied field. As can be seen in Figure 6, the FMP’s used in the tested suspensions exhibited a magnetic property in the presence of a magnetic field. Placing a magnet near the bottle containing magnetic suspensions (Figure 6), the FMP’s being attracted by a magnet (Figure 6A,C,D), and in the absence of the magnetic field, the Fe microparticles readily disperse in the suspensions by simple shaking (Figure 6A). In case of the absence of an external magnetic field, this model suspension is similar to a Newtonian fluid, but in the presence of an external magnetic field, the particles interact and form chain structures. These structures align along the direction of the magnetic field (Figure 6C,D), but in the absence of the magnetic field, no chain is observed.

Magneto-viscous characteristics were measured using a rotational rheometer (MCR 300, Physica, Stuttgart, Germany). Figure 5 shows that the Fe particle suspensions exhibit shear-thinning behavior in the absence of a magnetic field, probably due to the shear-thinning characteristics of the carrier fluid at low shear rates and because of the small aggregates of Fe microparticles that are progressively destroyed with shear intensification [42]. The rheological values for the aqueous glycerol solutions presented in Table 2 can be described using the Carreau model with four relevant parameters (Equation (1)) [43,44]:

(1) $$\eta \left(\dot{\gamma }\right)=\eta_{\infty }+\left(\eta_{0}-\eta_{\infty }\right){\left[1+{\left(C\dot{\gamma }\right)}^{2}\right]}^{-p},$$

where η_0_ is zero shear viscosity, η_∞_ is the viscosity at infinite shear rates, C is the characteristic time constant, and p is the flow behavior index. 

### 3.4. Magnetic Particle Characterization

In this study, Fe particles of 4–6 μm in size (Carl Roth GmbH, Karlsruhe, Germany) were used to model the magnetic carrier (Table 3). The used particles’ sizes are much larger than those used for drug delivery [45,46]. The results presented in the literature suggest that larger particles are better than smaller ones in terms of vessel wall margination, but larger particles, especially micro-sized ones, are potentially susceptible to physical entrapment in the capillaries [47,48,49]. 

Practically, this type of magnetic particle was chosen not for its commercial availability, but rather due to the results obtained by Pislaru et al. [40], who studied the endothelialization of 8 mm diameter synthetic vascular grafts (commercial Dacron grafts) using a 0.9 mm diameter coated iron oxide particle (a large diameter particle). On the other hand, we used this magnetic particle because its size is comparable to that of a red blood cell (7.5–8.7 μm).

It is mandatory to keep in mind that we used the 4–6 μm Fe particle as a model to demonstrate the feasibility of magnetic drug targeting in an artery bifurcation lesion (this type of particles is not intended to be used in clinical practice).

**Figure 7:**
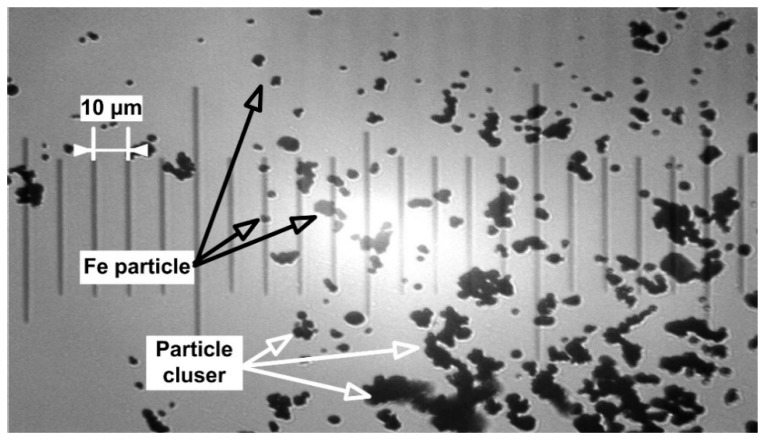
Microscopic image of the 4–6 μm Fe powder’s morphology. The image shows particle size distribution variation from the indicated mean size and the presence of the particle cluster.

The physical morphology of the used Fe particles was confirmed via a microscopic image presented in Figure 7.

**Figure 8:**
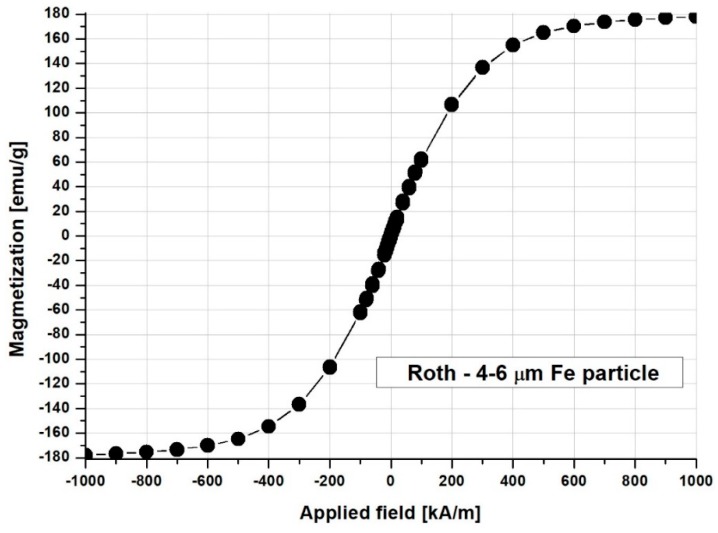
Magnetization curve and hysteresis loops measuring micro-sized (4–6 μm, Carl Roth GmbH, Karlsruhe, Germany) Fe particles in a powdered state. The hysteresis loops presented in the figure are smooth with no hysteresis, which indicates a soft magnetic material with coercive force, and the residual magnetization approaches zero.

Magnetic properties of the magnetic carrier (Fe particles) were measured using a vibrating sample magnetometer (VSM 880-ADE Technologies, USA) at room temperature (22 °C), with a field range of 0 kA/m to 950 kA/m. Figure 8 shows the full magnetization curves and hysteresis loops of the samples. As can be seen in Figure 8, the hysteresis loops are smooth with no hysteresis, which indicated a soft magnetic material with coercive force, and the residual magnetization approaches zero (Table 4).

### 3.5. Magnetic Field Generation

To achieve the best results for targeting, in this paper, the magnet positions were selected to analyze magnetic fields obtained both from numerical simulations and experimental measurements, placing permanent magnets at different distances from the targeted region.

The primary consideration during experiments is the rapid drop of the magnetic field due to the increase of the distance from the magnet’s surface [50], which restricts the targeting application to sites close to the magnetic sources (more so in the sites where the magnetic field can be safely applied) [51,52]. In terms of safety, in our experiment, the used permanent magnet produces a maximum magnetic field of 0.1 T–0.12 T. In the literature, the used magnetic field intensity depends on the applications (0.1 T–1.3 T for in vitro studies) [51,52,53] and remains below that of standard clinical MRI equipment (1.5 T–3 T) [54].

#### 3.5.1. The Experimentally Measured Magnetic Field

**Figure 9:**
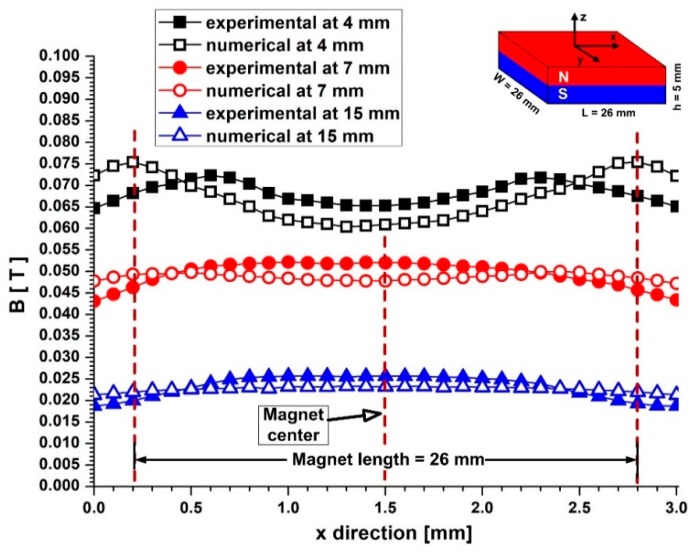
A magnetic field generated by the permanent ferrite magnet used in the experimental investigation. The ferrite magnet dimension and axis association: The used magnet has an axial polarization indicated in the figures; a comparison between the calculated and experimentally measured magnetic flux density produced by the ferrite type permanent magnet against the distance from the magnet surface (along axis Z). Experimental measurement was done using an F.W. Bell Gaussmeter, model 5080, and the magnetic field was obtained from numerical simulations using FEMM 4.2 software. The plot shows that the numerical solution of the magnetic field agrees well with the experimentally measured values. The differences are probably due to the precise position of the gaussmeter hole.

In the present study, the magnetic field was generated by a permanent ferrite type magnet, namely a Y8T ferrite magnet (commercial notation) with a maximum energy product (B × H) of 0.8–1.2 MGOe presented in Figure 9.

Ferrite magnets are also known as ceramic Magnets. Ferrite magnets are one of the most used permanent magnet materials in the world. Ferrite magnets are distinguished by their high magnetocrystalline anisotropic energy. Ferrite magnets are found in almost all industries, e.g., aerospace, electrical/electronic, military, automotive, sensor, advertising, academics, and R&D. 

Figure 9 shows the magnetic field induction B along with the different vertical distances from the surface (*z*-axis) of the used magnet, and a comparison between the experimental measurement and numerical simulation of the magnetic field for the same range of distances.

The spatial distribution of the magnetic field was measured using an F.W. Bell Gaussmeter, model 5080, with a 10 μT resolution and an accuracy of 1%. The vertical component of the resulting magnetic field is shown in Figure 9. As can be seen from the figure, the field intensity decreases rapidly from 0.08 T at y = 4 mm, to 0.02 T at y = 15 mm. The magnitudes of the field gradient components indicate the magnetic body force that the injected magnetic particles experience.

#### 3.5.2. Numerical Investigation of the Magnetic Field

**Figure 10:**
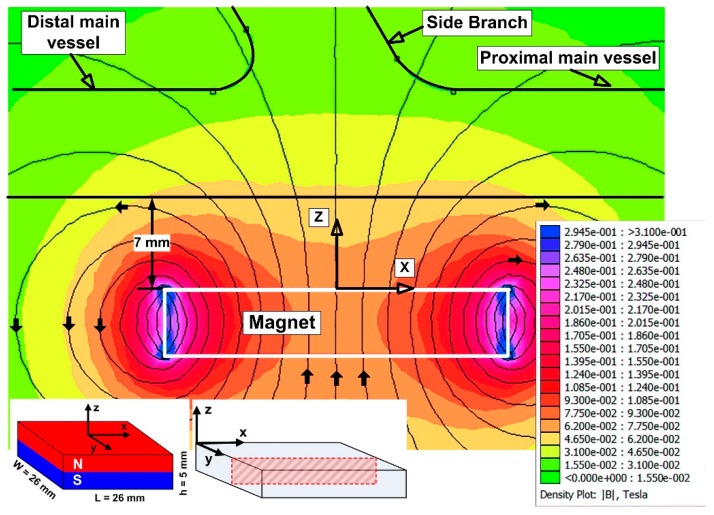
Numerical simulation of the magnetic field used during the experimental investigation (via the free Finite Element Methods Magnetics (FEMM) software). This figure shows the magnitude of the magnetic field produced by the Y8T permanent magnet in the longitudinal section, along with the bottom wall of the bypass graft at a distance of 7 mm from the magnet’s surface.

The magnetic field generated by the ferrite magnet was numerically investigated, using the freeware Finite Element Methods Magnetics (FEMM) software (http://www.femm.info/wiki/HomePage). The theoretical distribution of the magnetic field in the targeted region of the arterial bifurcation was investigated for a 2D problem (the longitudinal section of the used experimental setup) corresponding to the different vertical positions of the magnet from the targeted lesion (Figure 10). As can be seen from Figure 10, the permanent magnet generated a strong magnetic field in an open space (Figure 9 and Figure 10). When the distance between the permanent magnet and the vessel wall is small, the gradients of the magnetic field are high (>0.08 Tcm^−1^, Figure 9) (and higher in the vicinity of the dipole (>0.3 Tcm^−1^)). As a result, the injected MPs can be captured from the flow field in the targeted region and can build-up a stable deposition.

As expected, if the distance is considerable (small magnetic field gradients are present at <0.02 T), the chance of magnetic capture is small, and MPs are flushed out from the targeted region. Therefore, in practice, the fields become vanishingly small at sufficiently long distances from the given source.

## 4. Results and Discussions

### 4.1. Bifurcation Hemodynamics

In experiments, the local flow fields created bifurcations that were aligned with the specific hemodynamic environments along the wall of the real femoral artery bifurcation (Figure 1). 

**Figure 11:**
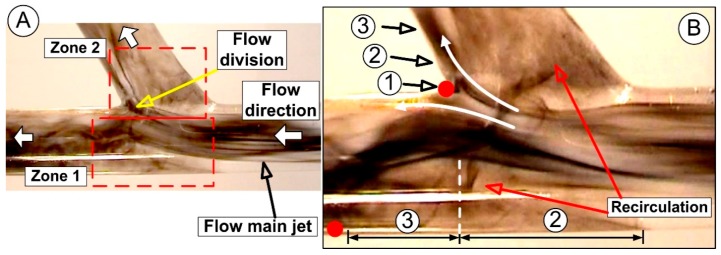
(**A**) The abnormal flow field is characterized by the flow division, strong flow impact around the apex, and flow oscillation on the host artery floor. The complex vortex structures created in the bifurcation area depend on the incoming flow structure; (**B**) detail of the flow bifurcation. Identification of the flow characteristic in the apex region: (1) impingement region, (2) acceleration region, (3) recovery region, and in the host artery floor: (2) acceleration zone and (3) deceleration (recovery) zone.

The presence of the low wall shear stress in this specific site can influence the behavior of endothelial cells, including expression of inflammatory cell attractors and morphological adaptations (inducing initialization and development of atherosclerosis, Figure 2) [29,40]. The difference between blood flow velocity in the center of the artery and the wall region, and the presence of the flow division around the bifurcation apex, generate a recirculation region both in the main vessel and its branches (Figure 11). It is essential to mention that platelet adhesion was localized within the recirculation region in the vicinity of the reattachment point (Figure 11). This is due to the enhanced transport of platelets to the arterial wall, along with the radially directed streamlines existing in the vicinity of the reattachment/stagnation point (Figure 11) [55]. These areas are characterized by impinging flow (the site where flow acts perpendicular to the bifurcation apex [56]), where the arterial wall experiences a high WSS level both in the apex and downstream of the impingement point [15] (Figure 2). 

As can be seen in Figure 11, the effects of flow separation at the bifurcations are the dominant factor on particle capture efficiency in the targeted site. 

The generated hemodynamic environment along the arterial wall of the bifurcation apex can be divided into three regions [55] for function of the flow evolution: (1) the impingement region, where flow division created a central stagnation point (Figure 11B), inducing flow acceleration in both branches (the main vessel and branches); (2) the acceleration region, where flow continued to accelerate along the branches wall (Figure 11B). From a medical point of view, this region is characterized by arterial wall remodeling due to the loss of the endothelial cells; and (3) the recovery region, characterized by the decreasing value of the wall shear stress until it returns to the baseline level (supplementary material: Video S1: flow structure in arterial bifurcation).

As the flow moves through the bifurcation segment in the distal direction, the luminal flow rate suffers flow division around the bifurcation apex. These luminal flow alterations induce extension and asymmetry of the recirculation region in the distal segment of the main bifurcating vessel. In this region, we found a strong velocity gradient between the main flow and the vortex structure in the vicinity of the arterial wall (Figure 11B). Furthermore, this region is characterized by wall remodeling due to the loss of endothelial cells.

### 4.2. Experimental Magnetic Particle Deposition

For particle accumulation in the targeted region, it is critical to understand the effect of the flow structure on deposition. In this paper the assumptions for the experimental investigations of the flow are: steady laminar flow, negligible gravitational effects, and constant physical properties.

#### 4.2.1. Principles of the Magnetic Particle Deposition in the Arterial Bifurcation

**Figure 12:**
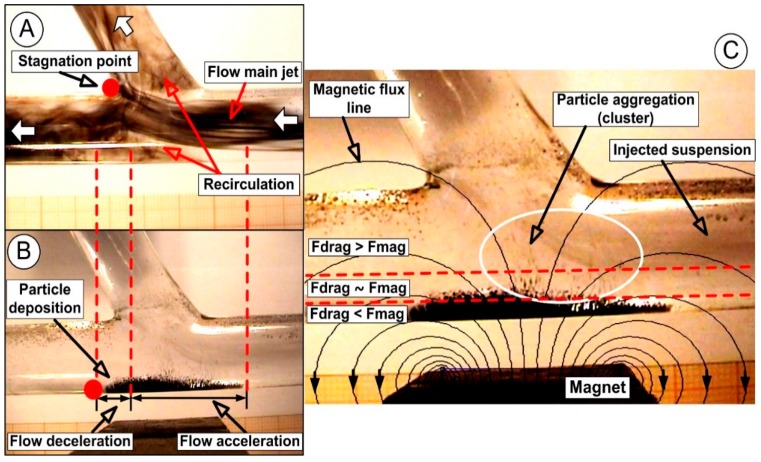
(**A**) Flow dynamics in the bifurcation region. (**B**) Particle deposition at the end of the injection time of 30 s. The distance between the permanent magnet and the bifurcation wall are 7 mm. (**C**) Magnetic and drag force distribution in the bifurcation region. The average intensity of the magnetic field along the bypass bottom wall was B = 0.05 T, according to Figure 9. In the case of comparable values of magnetic force and flow drag force, flow perturbation influences the FMP depositions, especially in the peripheral region.

In this investigation, hydrodynamic interactions between magnetic particles are negligible. Using ferromagnetic particle (FMPs) 4–6 μm diameter, the particles are essentially inertia-free, so the difference between the velocity of the flow around them and the velocity of the particle is negligible. As a result, ferromagnetic particle deposition occurs only where the magnetic force is too strong to counteract the drag force (Figure 12). Positioning a permanent magnet near to the diseased area obtained a deviation from the injected FMPs within the fluid flux and their capture onto the wall of the host vessels. 

In the present study, particle depositions were investigated for the following conditions (Table 5):

Before injection of the model suspension fluid, the samples were homogenized by mechanical stirring for 30s. After the sample reached the required homogeneity (checked visually; for an example, see Figure 6A), a quantity of 20 mL was injected into the fluid flow in front of the inlet section of the bifurcation model (using a syringe pump) for a duration of 30 s (injection time). Although the magnetic particles have a high density, the injection period of the suspension is low. Consequently, no particle sedimentation was observed inside the syringe pump.

During the experiments, a constant velocity profile flow was applied at the inlet section of the artery bifurcation model. The suspension of the magnetic particles was injected before the artery bifurcation inlet section. This suspension advected into the artery volume, creating a three-dimensional region of magnetic material. As a result, the generated magnetic force in the targeted region could slow and induce capture of the FMPs more successfully. 

Figure 12B,C shows that the fluid drag force acting on the FMP exceeds the magnetic forces generated by the permanent magnet. In this situation, blood velocity washes the particles downstream to the bifurcation region before the magnetic force affects the particles. 

The flow drag force on the particle varies with its position in the vessel. A particle at the vessel centerline will experience a higher velocity and hence a higher drag force, but a particle near the vessel wall will be surrounded by a low-velocity flow resulting in particles near the vessel wall experiencing a smaller drag force. In this situation, the trapped FMPs build up near the vessel wall, creating a specific shape of the deposition (Figure 12C). The deposition shape and the wash away occur due to the instabilities induced by the interaction between the flow structure, which generated a magnetic field, and particle deposition. Practically, only this kind of instability is responsible for the shape and size of the particle deposition in the wash away region. The significant consequence of this instability is the variable deposition shape, as can be seen in Figure 12 (different thicknesses for the acceleration and deceleration zones; supplementary video: Video S2: Particles deposition in arterial bifurcation).

#### 4.2.2. Particle Deposition

**Figure 13:**
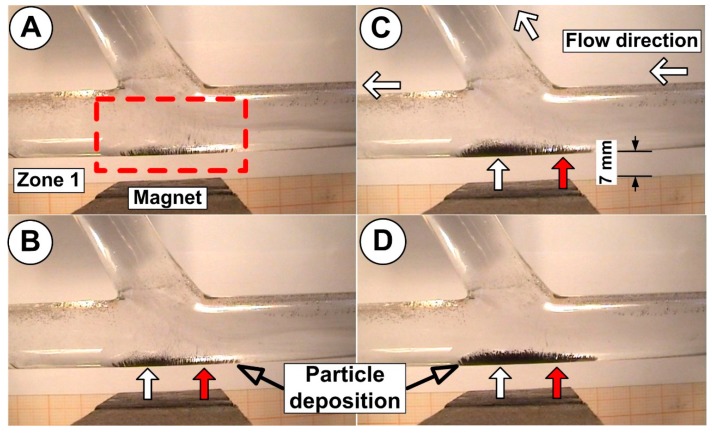
Particle retention in the artery bifurcation during an injection time of 30 s. The distance between the permanent magnet and the bifurcation wall are 7 mm. The average intensity of the magnetic field along the bypass bottom wall was B = 0.05 T, according to Figure 9. Particle deposition at different time steps: (**A**) T = 10 s, (**B**) T = 15 s, (**C**) T = 20 s, (**D**) T = 30 s (end injection). Different thicknesses of the particle deposition in the flow acceleration (red arrow) and deceleration regions (white arrow).

Because the distance between FMPs’ injection point and the bifurcation region is short, after a period of 2 s, the FMPs particle practically starts to deposit on the host tube wall (Figure 13A).

Figure 13 shows the variation in time of the FMP accumulation (during the injection period) in the bifurcation region (magnet position at 7 mm from the bifurcation bottom wall). For the investigated geometry, particles accumulated in low lying “dunes”, running across the entire pole face of the magnet, and slightly increased accumulation downstream from the stagnation point.

As can be seen in Figure 11, a complex flow structure was generated in the bifurcation area. The primary vortex is the more significant vortex closest to the apex, rotated anti-clockwise with a higher angular velocity. In this situation, the generated angular velocity induced fluid particles shedding from the central vortex into the main vessel flow (Figure 11).

The instabilities formed in the primary vortex disturbed the incoming flow from the bifurcation main vessel, inducing further fluctuations in the central vortex. Moreover, the presence of flow division around the apex increased the interaction between fluid flow, injected FMP particles, and generated the magnetic field. As a result, part of the injected particles is carried downstream both in the host vessel and the branches, and part of particles become trapped in the low shear stress regions of the main arterial wall (Figure 12).

Furthermore, the presence of the recirculation flow increases the particle near-wall residence time and induces particle deposition. Particle deposition shape is a result of the balance between the hydrodynamic force (drag force) generated by the angular velocity of the recirculation region and the magnetic field intensity generated by the permanent magnet (Figure 12 and Figure 13).

The main conclusion from this analysis refers to the fact that the horizontal and vertical position of the magnet is determinant for the quality of the accumulation in the targeted region (Figure 13).

As can be seen in Figure 13, the injected FMPs are attracted to the main vessel wall and have a very different distribution along the host arterial wall. Shape characteristics, length, width, and position, are directly correlated with the flow structure (stagnation point, recirculation, and flow structure, as presented in Figure 11) and the magnetic field intensity and field distribution (Figure 9). The shape length and thickness of particle deposition during the injection time are presented in Table 6.

**Figure 14:**
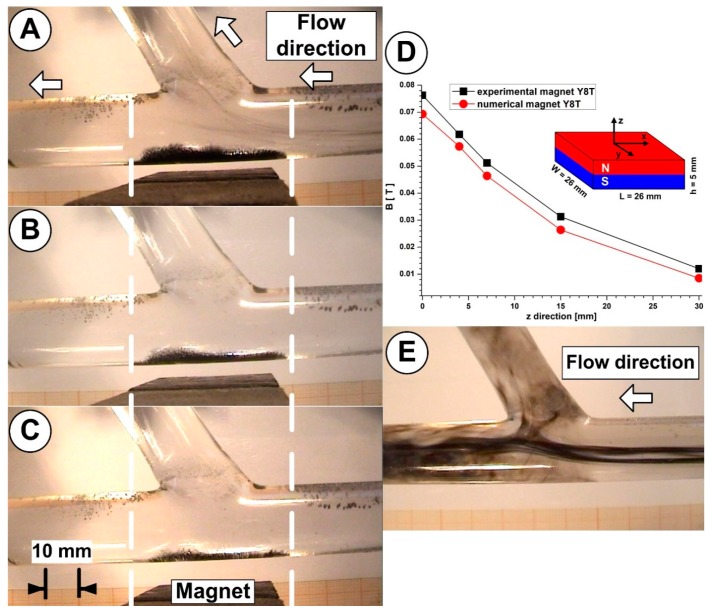
Particle accumulations in the bifurcation region for the different vertical positions of the magnet. (**A**) a magnet distance of 2 mm; (**B**) a magnet distance of 5 mm; (**C**) a magnet distance of 7 mm; (**D**) the magnetic field induction function of the distance to the magnet’s surface along the *z*-axis. The same working condition was applied for all magnet positions: an inlet velocity of 0.12 m/s, and an injection time of 30 s. (**E**) Flow evolution in the bifurcation section.

Figure 14 evidences the correlation between the particle deposition and the magnet position relative to the artery bifurcation wall. As can be seen in Figure 14, the particle deposition length is practically the same, but the shape of the deposition changes during the injection period. This, importantly, demonstrates that change in the method of accumulation lead to different amounts of trapped particles.

Quantitative correlations between magnet distance, magnetic field induction, and particle depositions (for the same working conditions described in Figure 14) for each investigated magnet positions are presented in Table 7.

### 4.3. Targeting Efficiency

Ferromagnetic particle deposition occurs only where the magnetic force is of sufficient strength to counteract the drag force (Figure 12). We define the targeting efficiency (TE) in the targeted area as the ratio between the FMP amount m_FMP_ accumulated in the targeted region and the total FMP amount m_total_ injected in the test section (Equation (2)):

(2) $$TE=\frac{m_{FMP}}{m_{total}}\times 100,$$

In our case, the m_FMP_ was determined experimentally from the weight difference between the injected amount of FMP in the suspension (m_total_ = 1 g) and the amount of the accumulated FMP in the targeted area. The accumulated FMP in the targeted area was collected after closing the flow system and removing the permanent magnet from the targeted area.

**Figure 15:**
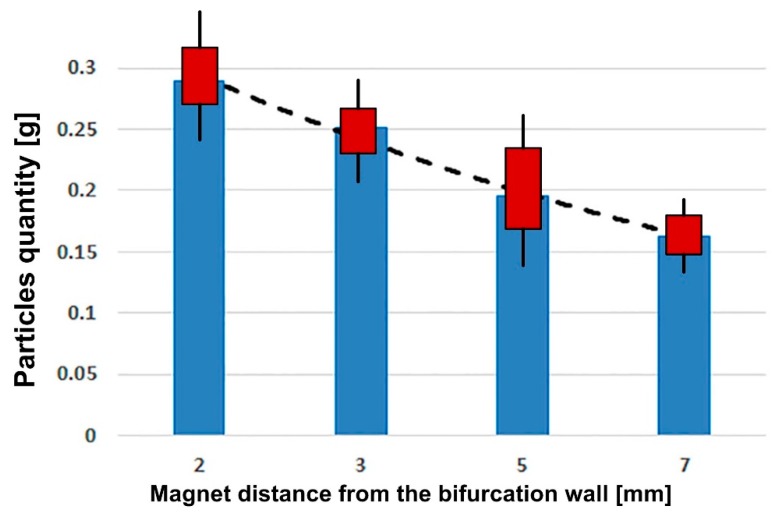
Particle accumulation efficiency in the targeted regions. Deposition trendlines are also represented.

To demonstrate the efficiency and the reproducibility of the targeting procedure, experimental measurements were repeated three times for each investigated magnet position in the same configuration (the same suspension concentration, same vessel bifurcation geometry, and same injection period). The targeting efficiency (TE) for the investigated cases is presented in Table 8 and Figure 15.

### 4.4. Medical Importance

In these experimental investigations, we used a steady flow condition. In steady flow, the fluid has a higher velocity compared to pulsatile flow. As a result, the fluid particle experienced a higher speed, and, consequently, the drag force increased, inducing a lower particle deposition. This conclusion depicts the importance of incorporating a more realistic arterial blood flow condition (pulsatile flow) into experimental magnetic drug targeting investigations.

On the other hand, it is essential to point out that the arterial wall is permeable. A permeable wall allows for a more easy capture of the targeted particle compared to an impermeable wall where some of the particles move away in the distal part of the bifurcation section. This is because the perfusing velocity (the axial velocity of the fluid) is lower in the permeable vessel than in the impermeable one [57].

In experimental processes, at the end of the particle injection period, the flow rate was turned off to filter and measure FMP depositions from the arterial bifurcation. Because this process is not instantaneous, some FMP particles were washed out from the targeted region. Practically speaking, the same phenomenon exists in medical practices during drug targeting.

Iron plays a critical role in various physiological functions and has an essential role in many metabolic processes, including oxidative phosphorylation, myelin synthesis, neurotransmitter production, such as dopamine and serotonin, and nitric oxide metabolism [58]. Furthermore, iron is an essential factor for the proper function of neurons [58]. Therefore, any disruption in the regulation of iron homeostasis can affect physiologic functions by increasing or decreasing the amount of iron in human cells [58].

Taking into account the results concerning the particle types and sizes used in medical practices (particle used for drug targeting), the Fe particles in the present investigations were used only to model the feasibility of the principle of magnetic drug targeting for more appropriate drug carriers (like PEG-coated iron oxide nanoparticles).

## 5. Conclusions

In the present paper, an in vitro flow model was established to examine and evaluate the efficiency of ferromagnetic particle accumulation inside the diseased artery bifurcation. The study results show that decreasing the distance between the permanent magnet and the targeted region lead to improved particle capture efficiency. It was ascertained that the location of the magnetic field source and the nature of the fluid flow are important factors influencing particle capture efficiency. 

Based on the experimental results, the following general features of the flow field were identified:

1. The flow bifurcation split flow structure was present in the main vessel and branch; both these structures had sizeable near-wall velocity fluctuations.

2. The active recirculation region is developed in the main artery and branches across the wall opposite to the bifurcation.

3. This recirculation region causes the particles to travel closer to the wall but, more importantly, increases particle residence time in this region. As a result, this region induces a more significant exchange of the mass transfer between the fluid and the vessel wall.

4. Particle targeting efficiency is correlated with the balance between the drag force and the magnetic force because the magnetic force is proportional to the cubed particle’s size and the particle’s hydrodynamic drag force is proportional to the particle’s size.

## 6. Future Steps

To improve this investigation’s results, a new type of carrier fluid will be used in future works, by introducing particles to mimic the presence and influence of red blood cells on blood hydrodynamics

**Figure 16:**
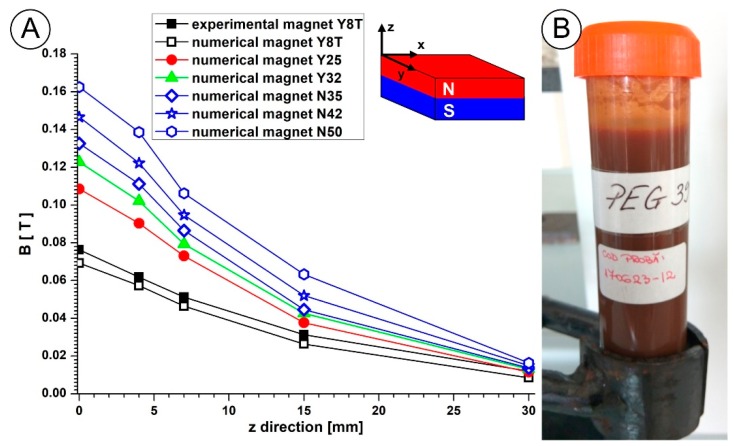
(**A**) Magnetic field induction function of the distance to the permanent magnet surface along the z axis for different types of magnets: the N35...52 Neodymium type magnet and the Y8T...Y32 ferrite type magnet. (**B**) Suspensions of the magnetic carrier containing PEG-coated nanoparticles.

For the next step of drug targeting efficiency investigations, two directions were identified: including a different type and size of permanent magnet in experimental investigations, and using a most appropriate magnetic carrier, like PEG-coated iron oxide nanoparticles. Our preliminary research indicated the feasibility of both directions (Figure 16A). Introducing a Neodymium type permanent magnet with a different energy product (B × H), it is possible to increase the depth of the targeted region (Figure 16). Regarding the magnetic carrier, many types of research have analyzed the advantages of biocompatible magnetic nanoparticles (MNPs) in drug targeting [59,60]. Recently, we investigated the rheological characteristics of a suspension containing multifunctional PEG-coated nanoparticles [61] for biomedical applications (Figure 16B). In the next step, we will investigate the potential of this carrier particle for targeting arterial bifurcation diseases.

As can be seen in Figure 16, ferrite type magnets are much weaker than rare earth magnets (NdFeB magnet). Typically, ferrite type magnets offer around 1/7th the pull force of similarly sized NdFeB magnets. The main advantages of Nd-Fe-B are their magnetic properties, high coercivity, and remanence. Regarding magnetic properties, the neodymium-iron-boron (NdFeB) permanent magnets can be purchased in strengths of up to 1.48 Tesla [62]. This is essential from a targeting point of view because this type of magnet can be used for particle targeting in blood vessels situated more profound in the body. For example, Lubbe et al. [63] used permanent magnets in human trials to target particles up to a 5 cm depth. Furthermore, larger targeting depths have been reported by Goodwin [64] (depths up to 12 cm) in animal experiments.

## Figures and Tables

**Table 1:** Particle trajectory visualization parameters.

Parameters	Values
Simulated artery bifurcation vessel inner diameter	8 mm
Blood velocity in the axial direction (Vmax)	0.12 m/s
Ferromagnetic particle diameters	4–6 μm
Magnetic field intensity (max)	0.15 T
Blood analog fluid viscosity	0.0036 mPa·s
The mass density of the ferromagnetic particles (Fe)	7680 kg/m^3^

**Table 2:** Rheological characterization of the carrier fluid and Fe particle suspensions.

Fluid	T [°C]	B [T]	$\eta_{\infty }[\mathrm{Pas}]$	$\eta_{o}[\mathrm{Pas}]$	C [s]	p [–]	r^2^
Carrier fluid - CF	25	0	0.0015	0.193	244.48	0.421	0.997
CF + 0.16% Fe	25	0	0.00154	0.095	18.08	0.664	0.999

Where: T [°C] is the carrier fluid temperature, B [T] is magnetic field intensity, η_∞_ [Pas] is viscosity at infinite shear rates, η_0_ [Pas] is zero shear viscosity, C [s] is characteristic time constant, and p [–] is flow behavior index. The r^2^ values for all fits are close to unity, indicating an excellent fit (r^2^ is the coefficient of determination used to evaluate quality of the Carreau fits).

**Table 3:** Fe particle characteristics.

Characteristics	Value
particle diameter	4–6 μm
density	7.86 g/cm^3^
molar mass	55.8 g/mol
chemical composition	in mass concentration percentage: Fe ≥ 99.5%; C ≤ 0.03%; O_2_ ≤ 0.2%; N_2_ ≤ 0.01%; Al ≤ 0.001%, As ≤ 0.0002%; Pb ≤ 0.0001%; Cu ≤ 0.001%; Mn ≤ 0.001%; Ca ≤ 0.001%; Cr ≤ 0.002%; Co ≤ 0.001%; Mg ≤ 0.001%.

**Table 4:** Fe particle magnetic properties (particles size of 4–6 μm).

Saturation Magnetization	Saturation Field	Coercive Field	Remanent Magnetization
Ms [A·m^2^/kg]: 177	Hs [kA/m]: 575	Hc [kA/m]: 1.32	Mr [A·m^2^/kg]: 0.891

**Table 5:** Particle deposition parameter setup.

Parameters	Particle Diameter	Mean Flow Velocity	Reynolds (Re)	Fluid Viscosity	Magnetic Field Induction
**Value**	4–6 μm	0.12 m/s	283	0.0036 Pa.s	0.07 to 0.15 T

**Table 6:** Characteristics of the particle accumulation shape during the injection time along the host’s arterial wall.

Time Step [s]	Accumulation Length [mm]	Average Thickness Corresponding to the Acceleration Zone [mm]	Average Thickness Corresponding to the Deceleration Zone [mm]	Magnetic Field Magnitude [T]
10	29	1	2.4	0.05
15	32	1.5	2.6	0.05
20	33	2	3.1	0.05
30	33	2.2	3	0.05

Effective quantitative measurements of particle accumulation are not performed for each time step.

**Table 7:** Characteristics of the particle accumulation for different magnet distances.

Magnet Distance [mm]	Magnetic Field Magnitude [T]	Accumulation Length [mm]	Particle Quantity [g]
2	0.068	36	0.289
5	0.058	35	0.195
7	0.048	33	0.163

**Table 8:** Targeting efficiency (TE) in the artery bifurcation model for different magnet distances.

Magnet Distance [mm]	Accumulated Quantity m_FMP_ [g]	TE [%]
7	0.163 ± 0.058	16.3
5	0.195 ± 0.085	19.5
3	0.251 ± 0.061	25.1
2	0.289 ± 0.072	28.9

## Supplementary Materials

The following are available online. Video S1: Flow structure in the arterial bifurcation; Video S2: Particle depositions in arterial bifurcation.
